# Predicting progress in word learning for children with autism and minimal verbal skills

**DOI:** 10.1186/s11689-021-09386-x

**Published:** 2021-09-15

**Authors:** Nancy C. Brady, Christine Kosirog, Kandace Fleming, Lindsay Williams

**Affiliations:** University of Kansas, 1000 Sunnyside Drive, 3000 Dole, Lawrence, KS 66045 USA

**Keywords:** Autism, Minimally verbal, Speech, Intervention

## Abstract

**Background:**

Approximately 30% of children diagnosed with autism remain minimally verbal past age 5. Interventions are often effective in increasing spoken communication for some of these children. Clinical and research decisions would be facilitated by identifying early indicators of progress in interventions. The purpose of this study was to investigate the relationship between speech sound measures obtained from the early phases of treatment and later treatment outcomes in children with autism and minimal verbal skills.

**Methods:**

Twenty-three children (18 boys) between 5 and 9 years of age participated. We compared scores reflecting the phonemic features of word attempts produced during probes, and the number of correct words after 4 weeks of intervention to later word learning outcomes.

**Results:**

Correlational and hierarchical regression analyses showed that both predictors were positively correlated with outcomes, but the phonemic scores were more strongly related than number of correct words.

**Conclusion:**

We conclude that phonemic scoring may be a useful measure to determine proximal gains in a spoken word learning intervention. Proximal measures are particularly helpful when trying to decide if the current course of intervention should be maintained or altered.

**Trial registration:**

https://register.clinicaltrials.gov/prs/app/action/LoginUser?ts=2&cx=-jg9qo3.

## Background

Approximately 25-30% of children diagnosed as having autism remain minimally verbal, speaking only a few words by age 5 [1–3]. Young children with autism who have not yet begun to talk may have intensive early language interventions available that focus on speech sound productions and receptive language training [4, 5]. Children age 5 and above, however, are more likely to have interventions that focus exclusively on augmentative and alternative communication (AAC) [6]. Recent efforts to teach spoken communication to children with autism past age 5 have had limited success [7, 8]. In these studies, some children respond to speech intervention by learning to produce new words, while others learn to say few, if any, words despite intensive interventions focused on speech production. The focus of the present study is to report on a measure designed to indicate proximal progress that may be predictive of expressive word learning. Specifically, we asked if a phonemic scoring measure during early phases of intervention would predict progress in learning to say new words by children with autism?

Most intervention studies aimed at teaching beginning speech and language skills to children with autism have focused on age 18 months to 5 years [5, 9, 10]. The focus is logical given that this is the age range during which the need for direct language intervention becomes apparent. In addition, intensive interventions are implemented early in hopes of preventing further gaps in language development. Despite the promise of intensive early interventions, approximately one-third of school-age children with autism do not use speech as a primary communication mode [11]. Remaining nonverbal past the age of 5 years is considered a poor prognostic indicator for future language development [12]. Although there have been reports of individuals older than age 5 acquiring speech (e.g., [12]), the characteristics of the successful individuals and the interventions employed are not fully understood.

Studies directly targeting speech sound productions in school-age children with autism and minimal expressive vocabularies have been rare. For example, Rogers and colleagues directly taught 5 preschool age participants speech skills using the PROMPT approach [13]. PROMPT is a method that provides kinesthetic information through touching to support motor control needed for articulation. All 5 children in the study by Rogers and colleagues increased their rate of word use per hour, but there were no experimental controls in place in this pilot study. King et al. [14] taught three participants to say target words and select AAC symbols representing the target words, on a speech generating AAC device. Children were between the ages of 4-8 and had severe speech-sound disorders not associated with autism. A multiple-baseline-across-participants design was used, and all 3 participants increased accurate productions of target vocabulary. Brady et al. [7] showed that a multimodal intervention that combined speech sound practice with AAC led to word learning by some school-age children with autism and minimal verbal skills. Chenausky et al. [8] found that an intervention focused on the intonational aspects of bisyllabic productions (e.g., cookie) improved word productions for some but not all their participants.

The interventions studied to date have been more effective with some children than others, a common finding in autism research. For example, Brady et al. [7] found that 5 of their participants responded well to intervention, learning many words in a few months. Three additional children showed gains but learned far fewer words and 2 children learned almost no words. Brady and colleagues found that children who, at the beginning of intervention, had relatively higher scores on verbal imitation, receptive vocabulary, communication scores on the Vineland, and nonverbal communication complexity measured with the Communication Complexity Scale (CCS, [15]) learned more words than participants with relatively lower scores in these areas. Verbal imitation serves as an indicator that the child is able to produce differentiated speech sounds in response to a modeled production.

The number of different sounds produced by children with autism and minimal verbal skills is also predictive of treatment outcomes. Chenausky et al. [16] found that the only significant predictor variable for improved syllable production, out of six potential predictors, was phonetic inventory, or the number of different sounds produced at intake. Similar findings were also reported by Saul and Norbury [17]. The current investigation examines an additional metric of speech productions derived from probes given during intervention. That is, rather than only considering initial speech productions prior to intervention, we evaluated early progress in an intervention aimed at improving spoken word productions using two possible predictors, the number of words learned during the initial stages of intervention and the phonemic scores for these word attempts. Our hypothesis was that, while both predictor measures are likely to be related to later learning outcomes, the phonemic scores would be more strongly related, and hence a more sensitive predictor. In addition, although participants were similar in terms of expressive language, we wanted to control for potential developmental differences that could also relate to outcomes. Our specific research questions are as follows:

1. Are two early learning measures, derived after 4 weeks of intervention, related to performance on subsequent word sets? The two measures are phonemic scores and number of words passed.
2. Does phonemic scoring at 4 weeks account for unique variance in performance on subsequent word sets beyond words passed at 4 weeks?
3. When we control for developmental differences between participants, does early phonemic performance still account for unique variance in performance on subsequent word sets?

## Methods

### Participants

Twenty-three participants (18 male) with autism participated in this study. The average age was 79.17 months with a standard deviation of 16.93 months. Recruitment occurred through flyers and consent forms distributed to parents within approved school districts, via special education teachers and speech language pathologists. Exclusion criteria included students learning English as a second language and any uncorrected vision or hearing impairments, severe motor impairments, or a dual diagnosis such as Down’s syndrome. The autism diagnostic observation schedule (ADOS [18]) was completed by a research-reliable provider to confirm diagnosis and document severity of autism symptoms. The comparison score on the ADOS provides a number from 1-10 indicating the level of autism spectrum-related symptoms present; a score of 1-2 indicates minimal to no evidence, 3-4 indicates low evidence, 5-7 indicates moderate evidence, and 8-10 indicates high evidence. The scores for participants ranged from 6-10, *M* = 7.4. At the time of the first data collection, all participants said fewer than 40 different words according to both parent and teacher report and none of the participants were putting words together into meaningful phrases. The Vineland Adaptive Behavior Scales-3 (VABS-3, [19]) was used to gather information on participants’ communication, social, daily living, and motor skills. It was completed online by participants’ classroom teachers. The mean adaptive behavior composite score was 54.73, SD = 9.37.

Students were participating in a randomized clinical trial of a multimodal word learning intervention. The clinical trial was paused due to COVID-19. Data for the current study were all obtained from the intervention phase of the study.

### Measures

#### Phonemic feature scoring

To better capture small gains or changes in speech production for minimally verbal participants, we implemented a phonemic feature scoring system. Each phoneme attempted by the participant during the probe was evaluated for accuracy as compared to the target consonant-vowel-consonant (CVC) word. For consonants, three features were measured: place of articulation, manner of articulation, and voicing. Place of articulation indicates where along the vocal tract a constriction is made to make a specific sound. Options include bilabial, labio-dental, dental, alveolar, palatal, velar, and glottal. Manner of articulation indicates how the speech articulators are used to produce the phoneme; options include stop, fricative, affricate, nasal, liquid, and glide. Voicing indicates whether the vocal folds are vibrating during the production of the sound (voiced consonants) or not (voiceless consonants). For vowels, four features were measured—height, advancement, rounding, tenseness. Height indicates where the tongue is during articulation and can be categorized by placement of low, mid, or high in the mouth. Advancement also refers to tongue placement, but from front to back of the mouth. Advancement can be categorized by tongue placement of front, back, or central. Rounding refers to rounding of the lips or not, while tenseness indicates whether the tongue is tensed or not (lax) during production of the vowel.

Each child production was evaluated according to these features. Each correct feature was given 1 point, for a total raw score of up to 10 points per word (3 for C1, 4 for V, 3 for C2). This means that the higher the total points, the closer the approximation is to the target word.

##### Examples of phonemic scoring

In example 1, the target CVC word is “bat.” In trial 1, the participant produces /baek/. For C1, they would receive 1 point each for voice, place, and manner for the correct production of /b/. For the vowel, they would receive 1 point each for height, advancement, rounding, and tenseness for the correct production of /ae/. For C2, the participant produces /k/ instead of /t/. As a result, they are given 1 point for voice, as both /k/ and /t/ are unvoiced. They are also given 1 point for manner because both the target consonant /t/ and the uttered consonant /k/ are stops. However, the participant is given 0 points for placement because the target /t/ is an alveolar while the uttered /k/ is a velar sound. In this case, the participant would receive a total of 9 points for trial 1.

In example 2, the target word is “cake.” In trial 1, the participant produces /dae/, leaving off C2 completely. For C1, the participant receives only 1 point for manner, as both /k/ and /d/ are stops. The participant is given 0 points for voice because /d/ is voiced and /k/ is not. They also receive 0 points for place because /k/ is a velar while /d/ is an alveolar sound. The vowel /ae/ is given 1 point for advancement and 1 point for roundness, as the target /e/ and the approximation /ae/ both share these phonemic features. For C2, the participant receives no points since that sound was not attempted at all. Thus, the total score for example 2 is 3 points.

#### Weekly probes

We applied the phonemic scoring system to child vocalizations during weekly probe sessions. For this, participants were asked to name pictures representing each of the 5 target words, comprising a word set. Each picture was presented 3 times in a random order for a total of 15 trials. Weekly probes continued for each set until the participant passed 3 of the 5 target words in 2 of 3 opportunities or 4 weeks elapsed, whichever came first. For our purposes, a score of at least 6 with the initial consonant correctly produced was considered a “passing” score for a word attempt.

Weekly probes were video recorded during the session and later transcribed in our lab. Two trained transcriptionists, blind to observation details including the week of the probe, and whether the participant was receiving intervention, transcribed each session. To optimize accuracy, each recording was first independently coded and then disagreements were resolved through consensus.

##### Scoring reliability

Two research assistants were trained to use the phonemic scoring system until they achieved a criterion of 85% agreement with videos previously coded by the fourth author, an experienced speech language pathologist. Subsequently, the research assistants each independently applied the phonemic scoring system to the transcribed data. A total of 3283 probes were coded by both research assistants. The intraclass correlation coefficient (ICC) for a two-way random effects model for absolute agreement of a single measure for these scores is .91. The ICC for average measures using the same model is .96. These coefficients indicate excellent reliability [20].

#### Vineland-3: Vineland Adaptive Behavior Scale-3 [19]

We used the composite adaptive behavior score as a control variable for developmental differences in our analyses. The Vineland Adaptive Behavior Scales-3 is administered individually by either a professional in an interview form, a parent/caregiver, or a teacher for ages birth to 91 years. For our purposes, we had teachers fill out the form, which has a narrower focus of ages 3-18 years. The form was completed online which provided information on the participant in a structured setting and allowed for more precise scoring using the online platform. The VABS-3 adaptive composite score consists of skills in communication, daily living skills, and socialization domains. The ABC standard scores are scaled so the mean is 100 within each age group and the standard deviation is 15. Averages of reliability for the teacher form fall in the excellent range.

### Data analyses

To address our research questions, we examined the associations between the scores from probes obtained from the end of the first 4 weeks of intervention to later scores. Our rationale for selecting week 4 was that this amount of time allowed participants to become familiar with the probe procedures. Probes were administered once per week. Hence, we were looking at early learning progress as an indicator of subsequent progress. Specifically, we examined the correlations between the following derived scores:

1. Week 4 phonemic score per trial (PS/TW4). The total phonemic score from the probe obtained at week 4 divided by the total number of trials (i.e., 15).
2. Week 4 words passed per trial (WP/TW4). The number of words passed during the week 4 session divided by the number of trials in the probe.
3. Phonemic score per trial for set 2 (PS/TS2). The total phonemic score for the best probe session during each word set that followed week 4 (set 2) divided by the number of trials in the probe. For example, if a participant’s best phonemic score during probes for set 2 was 17, this score was used in our analyses.
4. Words passed per trial set 2 (WP/TS2). The number of words passed during the best probe session of set 2, divided by the number of trials in the probe. For example, if a participant met our pass criterion for 5 words on their best probe for set 2, and there were 15 trials, their score for this variable would be .33. A score of 1.0 is the maximum for this variable.
5. Average words passed per week in all subsequent weeks (WP/WASW). The total number of words meeting our pass criteria for all sets after week 4 combined, divided by the total number of intervention weeks, after week 4.

For our research questions, we considered week 4 variables 1 and 2 above to be predictor variables and variables 3-5 as outcomes. Hierarchical regression models were then developed to examine the unique variance in each of the three outcomes accounted for by early phonemic scoring over and above early words passed.

## Results

Table 1 presents the descriptive information for each of the variables described above and used in our analyses.

**Table 1:** Descriptive information for variables used in our analyses

Variable	Minimum	Maximum	Mean	Standard deviation
Week 4 phonemic score per trial	.07	9.0	3.41	2.65
Week 4 words passed per trial	.00	.87	.20	.27
Phonemic score per trial for set 2	.27	9.47	5.25	3.08
Words passed per trial set 2	0	1	.426	.378
Average words passed per week subsequent weeks	0	8.13	3.23	2.68
VABS 2 composite	40	74	55.42	9.36

### Research questions 1 and 2

1. Are phonemic scoring and words passed derived after 4 weeks of intervention related to performance on subsequent word sets?
2. Does phonemic scoring at 4 weeks account for unique variance in performance on subsequent word sets beyond words passed at 4 weeks?

**Fig. 1:**
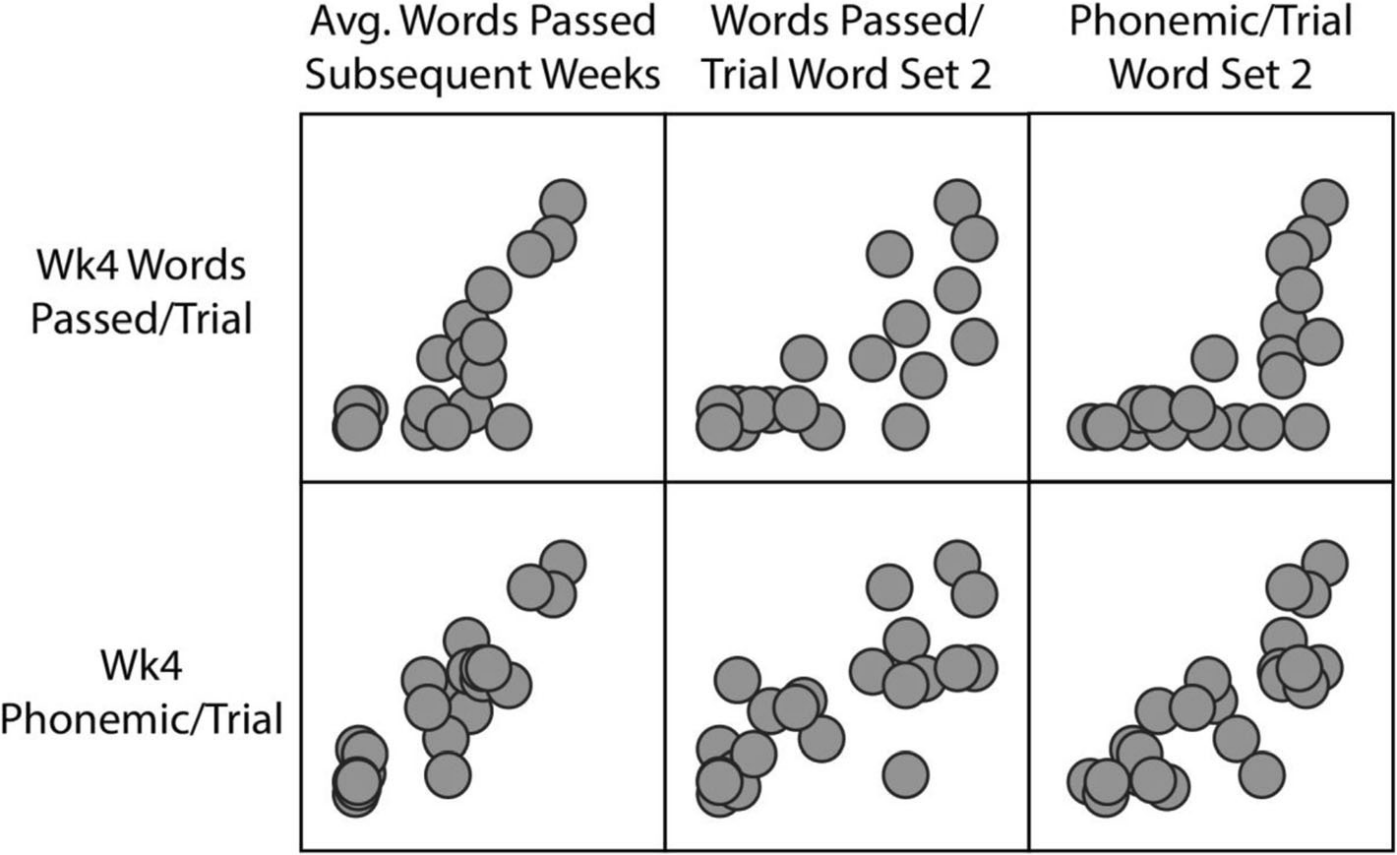
Scatterplots of week 4 words passed per trial, week 4 phonemic score per trial, and the three outcomes

We first ran correlations between variables to identify relationships (see Table 2 and Fig. 1). As can be seen in Table 2, the highest correlation was between the week 4 phonemic score per trial and the average words passed per week subsequent weeks. Figure 1 presents a scatterplot of week 4 words passed per trial, week 4 phonemic score per trial, and the three outcomes. As can be seen in Fig. 1, more scores for the week 4 words passed per trial were at the floor, and more variability was detected with the week 4 phonemic scores per Trial.

**Table 2:** Correlations between week 4 phonemic scores per trial and week 4 words passed per trial and outcome measures

	Week 4 phonemic score per trial	Week 4 words passed per trial	Phonemic score per trial for set 2	Words passed per trial for set 2
Week 4 words passed per trial	.878**			
Phonemic score per trial for set 2	.818**	.692**		
Words passed per trial set 2	.773**	.730**	.952**	
Average words passed per week subsequent weeks	.911**	.776**	.910**	.894**

Note: *p < .05. **p < .01. ***p < .001

Next, we ran hierarchical regression models for each of the three outcome variables, phonemic score per trial for set 2, words passed per trial for set 2, and average words passed per week in all subsequent weeks (see Table 3). For each outcome, the first model included week 4 words passed per trial as a predictor. In the second model, week 4 phonemic score per trial was added as a second block. The overall *R*^2^ from model 2 for each outcome addressed research question 1, and the significance of the phonemic score per trial in week 4 predictor from model 2 addressed research question 2.

**Table 3:** Hierarchical regression analysis summary for 3 dependent variables—phonemic score/trial set 2, words passed/trial set 2, and words passed/week all subsequent weeks

	***R***^**2**^	Δ ***R***^**2**^	Δ***F***	DF	***β***	SE ***β***	Prob ***β***	Part
**DV—phonemic score/trial set 2**								
**Model 1**	.48	.48	19.28***	(1,21)				
Week 4 words passed/trial					.69	.49	.001	.69
**Model 2**	.67	.19	11.81**	(1,20)				
Week 4 words passed/trial					−.12	.83	.668	−.06
Week 4 phonemic/trial					.92	.83	.003	.44
**DV—words passed/trial set 2**								
**Model 1**	.53	.53	23.99***	(1,21)				
Week 4 words passed/trial					.73	.06	.001	.73
**Model 2**	.61	.08	3.90^	(1,20)				
Week 4 words passed/trial					.22	.11	.454	.11
Week 4 phonemic/trial					.58	.11	.062	.28
**DV—words passed/week all subsequent weeks**								
**Model 1**	.60	.60	31.88***	(1,21)				
Week 4 words passed/trial					.78	.37	.001	.78
**Model 2**	.83	.23	27.20***	(1,20)				
Week 4 words passed/trial					−.10	.51	.599	−.05
Week 4 phonemic/trial					1.00	.51	.001	.48

*** = < .001, ** = < .01, * = < .05, ^ = < .10

As indicated by the Model 2 *R*^2^ values presented in Table 3, we found that early indicators of week 4 words passed per trial and week 4 phonemic score per trial accounted for significant variance in all three outcomes. The two predictors accounted for 83% of the variance in average words passed per week in all subsequent weeks, 67% of the variance in phonemic score per trial for set 2, and 61% of the variance in words passed per trial set 2.

The initial model, regressing PS/TS2 on WP/TW4, was significant, *F* (1,21) = 19.28, *p* < .001. WP/TW4 accounted for 48% of the variance in PS/TS2. However, when PS/TW4 was added as a predictor, it uniquely accounted for 19% of the variance (*p* = .003) and WP/TW4 uniquely accounted for less than 1% of variance (part correlation squared = .003, *p* = .67). The initial model, regressing WP/TS2 on WP/TW4, was significant, *F* (1,21) = 23.99, *p* < .001. WP/TW4 accounted for 53% of the variance in WP/TS2. However, when PS/TW4 was added as a predictor, it uniquely accounted for 8% of the variance (*p* = .06) and WP/TW4 uniquely accounted for a nonsignificant 1% of variance (part correlation squared = .01, *p* = .45). The initial model, regressing WP/WASW on WP/TW4, was significant, *F* (1,21) = 31.88, *p* < .001. WP/TW4 accounted for 60% of the variance in WP/WASW. However, when PS/TW4 was added as a predictor, it uniquely accounted for 23% of the variance (*p* < .001) and WP/TW4 uniquely accounted for less than 1% of variance (part correlation squared = .01, *p* = .45).

### Research question 3

When we control for developmental differences between participants, does early phonemic performance still account for unique variance in performance on subsequent word sets?

Hierarchical linear regression models with Vineland Composite Standard Scores entered as block 1, week 4 words passed per trial as block 2, and week 4 phonemic score per trial as block 3 were also examined (see Table 4). Results were similar to the other hierarchical models with the two predictors accounting for slightly less overall variance, *R*^2^s of .76, .62, and .56, respectively.

**Table 4:** Hierarchical regression analysis summary for three dependent variables with Vineland covariate

	***R***^**2**^	Δ ***R***^**2**^	Δ***F***	DF	***β***	SE ***β***	Prob ***β***	Part
**DV—phonemic score/trial set 2**								
**Model 1**	.04	.04	.68	(1,17)				
Vineland total SS					.20	.69	.422	.20
**Model 2**	.40	.36	9.62**	(1,16)				
Vineland total SS					−.01	.59	.968	−.01
Week 4 words passed/trial					.64	.68	.007	.60
**Model 3**	.62	.22	8.92**	(1,15)				
Vineland total SS					−.09	.49	.622	−.08
Week 4 words passed/trial					−.03	.93	.928	−.02
Week 4 phonemic/trial					.84	.92	.009	.47
**DV—words passed/trial set 2**								
**Model 1**	.07	.07	1.36	(1,17)				
Vineland total SS					.27	.08	.260	.27
**Model 2**	.47	.40	11.94**	(1,16)				
Vineland total SS					.06	.07	.764	.06
Week 4 words passed/trial					.66	.08	.003	.63
**Model 3**	.56	.09	3.02	(1,15)				
Vineland Total SS					.01	.06	.956	.01
Week 4 words passed/trial					.25	.12	.420	.14
Week 4 phonemic/trial					.53	.12	103	.30
**DV—words passed/week all subsequent weeks**								
**Model 1**	.10	.10	1.90	(1,17)				
Vineland total SS					.32	.56	.186	.32
**Model 2**	.47	.37	11.08**	(1,16)				
Vineland total SS					.11	.47	.573	.11
Week 4 words passed/trial					.64	.54	.004	.61
**Model 2**	.76	.30	18.73***	(1,15)				
Vineland total SS					.02	.33	.867	.02
Week 4 words passed/trial					−.12	.62	.601	−.07
Week 4 phonemic/trial					.96	.62	.001	.54

*** = < .001, ** = < .01, * = < .05, ^ = < .10

Week 4 phonemic scores per trial uniquely accounted for slightly more variance in average words passed subsequent weeks (30%) and phonemic score set 2 per trial (22%) as compared with models not controlling for developmental differences.

## Discussion

Interventions for children with autism and minimal verbal skills are typically time and resource intensive. It can be difficult to determine if interventions are leading to improvement because progress may be slow or sporadic. In the case of speech interventions, progress may be particularly difficult to measure if children are struggling to accurately produce words, and accurate word production is the only index of progress. Progress may be demonstrated more proximally by examining changes in the sounds that comprise words. Changes in syllables and phonemes may occur before progress is detected at the word level. There are currently few guidelines available for researchers and clinicians to determine if proximal changes in these components of words are indicative of early progress in speech. The current study addressed this gap by examining the relationships between scores derived from a phonemic scoring system to progress in a spoken word intervention.

The phonemic scoring system is designed to be sensitive to subtle changes in speech sound productions. The scoring system requires practice and knowledge about how phonemes are produced. Hence, it is not as easy to apply as other speech sound indicators such as phonemic inventories. This increased time required for phonemic scoring could be worth it if it yields valuable information about changes that are not detected elsewise and can be used to inform intervention practice. For example, a student may show very slow progress in producing accurate words during intervention, but a check after a few weeks indicates progress in word approximations evidenced in phonemic scoring. In such a case, the clinician may decide to continue the current intervention or to make slight adjustments to address some persistent errors. Other students may show little progress on words and sound productions. A pattern such as this would suggest a different type of intervention may be needed.

We found that phonemic scores at week 4 accounted for more unique variance in outcomes than the number of words passed at week 4. This is noteworthy because it suggests that measuring speech sound productions has value in detecting early progress. In addition to indicating if a student or research participant is “on the right track” or not, phonemic scores may be useful for detecting speech components to target. For example, vowels may be particularly problematic and lead to overall word production errors. Similarly, persistent final consonant deletion could further impact word scores and intelligibility. Each of these error patterns could be addressed through more prescriptive interventions.

### Strengths and limitations

We were able to show that a phonemic scoring system is strongly related to word production outcomes in children with autism and minimal verbal skills, and that the relationships are stronger than relationships between word production scores and outcomes. The scoring system was reliable, and our results remained even after we controlled for developmental differences.

The phonemic scoring system used in this project has been shown to correlate with listener judgments of intelligibility in an earlier study with eight participants [21]; however, this finding needs to be replicated with a larger sample. In addition, developing methods to make judgments about phoneme accuracy from live observations would significantly improve the feasibility of applying phonemic scoring to clinical procedures.

### Future directions

Future investigations will determine if the phonemic scoring described in this paper is specifically linked to intervention effects. In addition, we will determine if there is added value for phonemic scoring above the phonemic inventories and other predictor variables. These analyses require the conclusion of the currently paused clinical trial.

Future studies may also address the feasibility factor by comparing scores developed for, and used in, the current study, to others that may be implemented live in the field. Such efforts are important for continuing to develop effective interventions for children with minimal verbal skills and autism.

## Conclusions

Children aged 5 and above with autism and minimal verbal skills require intensive interventions to improve speech production. Research in expressive speech development is sparce and it can be difficult to measure progress in learning to produce spoken words, particularly progress in producing the speech sounds that comprise target words. The phonemic scoring system offers a way to measure small changes in expressive speech and can be used to determine the next steps in intervention. Our results indicate that the phonemic scoring system shows a strong relationship to word production outcomes and is reliable in its use. The phonemic scoring system holds promise as a proximal indicator of progress in future intervention studies targeting spoken words.

## Data Availability

The datasets used and/or analyzed during the current study are available from the corresponding author on reasonable request.
